# Tailoring therapies to counter the divergent immune landscapes of breast cancer

**DOI:** 10.3389/fcell.2023.1111796

**Published:** 2023-02-22

**Authors:** Sherif Attalla, Tarek Taifour, William Muller

**Affiliations:** ^1^ Department Biochemistry, Faculty of Medicine and Health Sciences, McGill University, Montreal, QC, Canada; ^2^ Goodman Cancer Institute, Faculty of Medicine and Health Sciences, McGill University, Montreal, QC, Canada; ^3^ Department Experimental Medicine, Faculty of Medicine and Health Sciences, McGill University, Montreal, QC, Canada

**Keywords:** breast cancer, immunology, immune microenvironment, therapies, and clinical trials

## Abstract

Breast cancer remains a significant clinical concern affecting millions of women worldwide. Immunotherapy is a rapidly growing drug class that has revolutionized cancer treatment but remains marginally successful in breast cancer. The success of immunotherapy is dependent on the baseline immune responses as well as removing the brakes off pre-existing anti-tumor immunity. In this review, we summarize the different types of immune microenvironment observed in breast cancer as well as provide approaches to target these different immune subtypes. Such approaches have demonstrated pre-clinical success and are currently under clinical evaluation. The impact of combination of these approaches with already approved chemotherapies and immunotherapies may improve patient outcome and survival.

## Introduction

**FIGURE 1:**
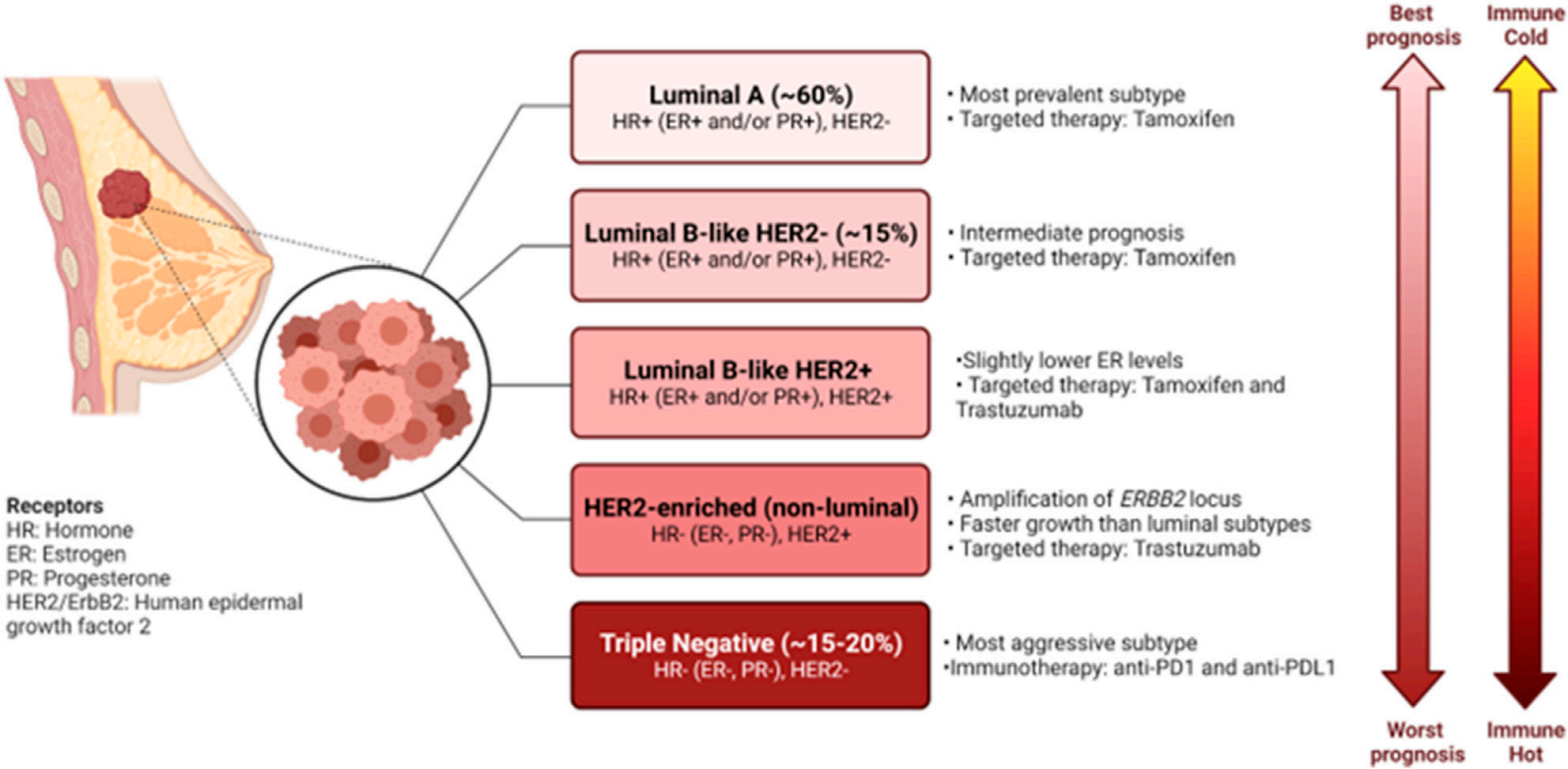
Characteristics of the molecular subtypes of breast cancer. Breast cancers can be classified based on gene expression profiles and receptor status. Each of the subtypes exhibit their own clinical manifestations, disease outcome, treatment options and various immune profile. Figure generated with biorender.com.

Breast cancer remains one of the major causes of death of women, with an estimated 15 Canadian women succumbing to the disease everyday in 2022 (Canadian Cancer Society, 2022). Despite the great progress made in early detection and treatment of breast cancer, it remains the second largest cause of death due to cancer in women (DeSantis et al., 2019; Harbeck et al., 2019). Moreover, almost all cases of death due to breast cancer are caused by metastases to essential organs (Harbeck et al., 2019). Breast cancers are classified according to a combination of molecular and histological subtypes (Harbeck et al., 2019). Tumors are classified as hormone receptor positive (HR+), human epidermal growth factor 2 positive (HER2+) and triple negative breast cancers (TNBCs) (Harbeck et al., 2019). These molecular subtypes display diverse clinical manifestations, disease outcome, treatment options and various immune profiles (Harbeck et al., 2019; Onkar et al., 2023) (Figure 1). It has become clear that the intra- and inter-tumor heterogeneity of breast cancer drives formation of various tumor immune microenvironments (TIMEs), that have become critical in prediction of responses to immunotherapy (Hammerl et al., 2021).

Immunotherapies have demonstrated strong potential in tumors such as melanoma (Huang and Zappasodi, 2022), however, only remain marginally successful in breast cancers (Schmid et al., 2020), arguing that a better understanding of the diverse immune landscapes of breast cancers and thus tailoring therapies to those tumors is required to improve patient outcome.

In this review, we aim to emphasize how the immune microenvironment may be used for clinical classifications of tumors and how the TIME regulates breast cancer growth, dissemination, and metastasis. These studies highlight immune mediated pathways that are either under clinical evaluation or may provide clinically relevant targets for future therapies (Table 1). We focused exclusively on discoveries made in the context of breast cancer as they represent a unique tumor model arising in mainly females and thus are responsive to estrogens.

**TABLE 1:** Summary of the clinical trials mentioned in this paper. Ordered based on appearance in the text.

Trial code	Phase	Status	Agent tested	Condition
**NCT05070247**	I	Recruiting	TAK-500 (STING agonist) alone or in combination with Pembrolizumab (anti-PD1)	Pancreatic, hepatocellular, breast, gastric, esophageal cancers as well as squamous cell cancers, non-small cell lung cancers and mesothelioma
**NCT04144140**	I	Completed	E7766 (STING agonist)	Advanced solid cancers and lymphomas
**NCT03956680**	I	Active	BMS-986301 (STING agonist) alone or in combination with Nivolumab (anti-PD1) and Ipilimumab (anti-CTLA4)	Advanced solid cancers
**NCT03843359**	I	Recruiting	GSK3745417 (STING agonist) alone or in combination with Dostarlimab (anti-PD1)	Advanced solid cancers
**NCT03010176**	I	Completed	Ulevostinag (STING agonist) alone or in combination with Pembrolizumab (anti-PD1)	Solid tumors and lymphoma
**NCT03249792**	I	Active	MK-2118 (STING agonist) alone or in combination with Pembrolizumab (anti-PD1)	Solid tumors and lymphoma
**NCT04096638**	I	N/A	SB 11285 (STING agonist) alone or in combination with Atezolizumab (Anti-PDL1)	Solid tumors, head and neck cancers and melanoma
**NCT03616886**	I/II	Active	Oleclumab (also known as MEDI9447, an anti-CD73 antibody) in combination with Paclitaxel and Carboplatin (chemotherapy) as well as Durvalumab (anti-PD1). Control cohort will receive Paclitaxel, Carboplatin and Durvalumab	Triple negative breast cancers
**NCT03875573**	II	Recruiting	Paclitaxel and dose-dense doxorubicin-cyclophosphamide (chemotherapy) with or without Oleclumab (anti-CD73 antibody) or Durvalumab (anti-PD1)	Luminal B breast cancers
**NCT03454451**	I	Active	CPI-006 (anti-CD73 antibody) alone, in combination with Ciforadenant (antagonist for adenosine 2A receptor), in combination with Pembrolizumab (anti-PD1) or combined with both Ciforadenant and Pembrolizumab	Renal cell, colorectal, cervical, ovarian, pancreatic, endometrial and bladder cancers as well as TNBCs, non-small cell lung cancers, sarcomas, squamous cell carcinoma of the head and neck, metastatic castration resistant prostate cancers and non-Hodgkin lymphoma
**NCT05431270**	I	Recruiting	PT199 (anti-CD73 antibody) alone or in combination with anti-PD1	Advanced solid tumors
**NCT05143970**	I	Recruiting	IPH5301 (anti-CD73) alone or in combination with Paclitaxel (chemotherapy) and Trastuzumab (anti-HER2 antibody)	Advanced solid and metastatic cancers
**NCT05001347**	II	Active	TJ004309 (anti-CD73) combined with Atezolizumab (Anti-PDL1)	Ovarian, head and neck, gastrointestinal, non-small cell lung cancers and TNBCs
**NCT04148937**	I	Completed	LY3475070 (selective CD73 inhibitor) alone or in combination with Pembrolizumab (anti-PD1)	Advanced cancers
**NCT03978663**	N/A	Recruiting	Neoadjuvant radiation	High risk cancers and locally advanced breast cancers
**NCT01803503**	II	N/A	Docetaxel (chemotherapy) with or without pre-treatment with Sunitinib (angiogenesis inhibitor)	Solid tumors, Breast, prostate, gastric and non-small cell lung cancers
**NCT03184558**	II	Terminated	Bemcentinib (AXL inhibitor) in combination with Pembrolizumab (anti-PD1)	TNBC and inflammatory breast cancers
**NCT04842812**	I	Recruiting	CAR-T cells against various tumor antigens (Ex: HER2, AXL, EGFR and others)	Liver, lung, breast, colorectal and brain cancers
**NCT04355858**	II	Recruiting	Depending on the patient’s molecular profile, different therapeutics are administered	HR+ HER2- endocrine resistant breast cancer
			• Patients with NF1 mutation: SHR7390 (MEK 1/2 inhibitor) in combination with Famitinib (receptor tyrosine kinase inhibitor)	
			• Patients with gBRCA mutation: SHR3162 (PARP inhibitor) in combination with SHR6390 (CDK4/6 inhibitor)	
			• Patients with HER2 activating mutations: Pyrotinib (HER1/2 receptor tyrosine kinase inhibitor) in combination with Capecitabine (chemotherapy)	
			• Patients with PDGFRb mutation: Famitinib (receptor tyrosine kinase inhibitor)	
			• Patients with evidence of marked CD8 T-cell presence (≥10% by IHC) will receive one of the following therapeutics based on their medical history: Famitinib (receptor tyrosine kinase inhibitor) or SHR1210 (anti-PD1), Nab paclitaxel (chemotherapy), SHR6390 (CDK4/6 inhibitor), SHR1701 (anti-PDL1/TGFβRII bifunctional fusion protein), Fulvestrant (selective estrogen receptor degrader), Aromatase inhibitor, or Bevacizumab (VEGF inhibitor)	
			• Patient with a PAM pathway mutation: Everolimus (mTOR inhibitor) in combination with Nab paclitaxel (chemotherapy)	
			• Patient expresses Androgen receptor (AR≥10% by IHC): SHR2554 (EZH2 inhibitor) and SHR3162 (PARP inhibitor)	
**NCT03805399**	I/II	Recruiting	Depending on the patient’s subtype, different therapeutics are administered	TNBC.
			• Luminal androgen receptor (LAR) subtype with HER2 activating mutations: Pyrotinib (HER1/2 receptor tyrosine kinase inhibitor) in combination with Capecitabine (chemotherapy)	
			• LAR subtype with a PIK3CA mutation but without HER2 activating mutations: SHR3680 (AR inhibitor) combined with Everolimus (chemotherapy)	
			• LAR subtype without PI3KCA nor HER2 activating mutations: SHR3680 (AR inhibitor) combined with SHR6390 (CDK4/6 inhibitor) or SHR2554 (EZH2 inhibitor)	
			• Immunomodulatory (IM subtype): SHR1210 (anti-PD1) combined with Nab-paclitaxel (chemotherapy)	
			• Basal-like immune suppressed (BLIS) with a BRCA mutation: SHR3162 (PARP inhibitor) combined with Famitinib (receptor tyrosine kinase inhibitor)	
			• Basal-like immune suppressed (BLIS) without a BRCA mutation: VP-16 (chemotherapy) in combination with Apatinib (VEGFR2 inhibitor) or Famitinib (receptor tyrosine kinase inhibitor). Patients may alternatively receive BP102 (VEGF inhibitor) in combination with Nab-paclitaxel (chemotherapy)	
			• Mesenchymal (MES) subtype without PI3K/AKT pathway activation: Famitinib (receptor tyrosine kinase inhibitor) in combination with VP-16 (chemotherapy)	
			• Mesenchymal (MES) subtype with PI3K/AKT pathway activation: mTOR inhibitor in combination with Nab-paclitaxel (chemotherapy)	
**NCT05600582**	I	Not yet recruiting	CodaLytic (oncolytic virus)	Metastatic or inoperable Breast cancers
**NCT05081492**	I	Recruiting	CF33-hNIS-antiPDL1 (oncolytic virus)	Stage 4 breast cancer or metastatic TNBC.
**NCT03004183**	II	Active	ADV/HSV-tk (oncolytic virus) in combination with Valacyclovir (anti-viral), stereotactic body radiation therapy and Pembrolizumab (anti-PD1)	Metastatic TNBC and metastatic non-small cell lung cancers
**NCT04445844**	II	Recruiting	Pelareorep (Oncolytic virus) in combination with Retifanlimab (anti-PD1)	Stage 4 breast cancer, metastatic TNBC and locally advanced breast cancers
**NCT02779855**	I/II	Active	Talimogene laherparepvec (Oncolytic virus) in combination with Paclitaxel (chemotherapy)	Breast cancer
**NCT04185311**	I	Active	Talimogene laherparepvec (Oncolytic virus) in combination with Nivolumab (anti-PD1) and Ipilimumab (anti-CTLA4)	Breast cancer of any stage except stage 4
**NCT05076760**	I	Recruiting	MEM-288 (oncolytic virus)	Solid or metastatic cancers, non-small cell lung cancer, cutaneous squamous cell carcinoma. Merkel cell carcinoma, melanoma, TNBC, head and neck and pancreatic cancers
**NCT04215146**	II	Active	Pelareorep (oncolytic virus) in combination with Paclitaxel (chemotherapy) or Paclitaxel and Avelumab (anti-PDL1)	Metastatic breast cancers
**NCT03740256**	I	Recruiting	CAdVEC (oncolytic virus)	Bladder, salivary gland, lung, breast, gastric, esophageal, colorectal cancers, head and neck squamous cancers and pancreatic adenocarcinoma
**NCT05180851**	I	Recruiting	Recombinant L-IFN adenovirus (oncolytic virus)	Melanoma, head and neck, breast, bladder, ovarian, cervical and lung cancers
**NCT04521764**	I	Recruiting	Oncolytic Measles Virus Encoding *Helicobacter pylori* Neutrophil-activating Protein (MV-s-NAP)	Stage 4, metastatic and recurrent breast cancers
**NCT05378464**	I	Recruiting	Adoptive T-cell therapy following dendritic cell vaccination (DC1) in combination with Trastuzumab (anti-HER2 antibody) and Pepinemab (anti-SEMA4D antibody)	HER2+ breast cancers
**NCT02778685**	II	Suspended	Palbociclib (CDK4/6 inhibitor) and Pembrolizumab (anti-PD1) in combination with Fulvestrant (selective estrogen receptor degrader) or Letrozole (aromatase inhibitor)	Stage 4 or metastatic breast cancer
**NCT03573648**	II	Recruiting	Patients will receive endocrine therapy alone or in combination with Palbociclib (CDK4/6 inhibitor)	ER+ Breast cancers
			For pre-menopausal women, endocrine therapy consists of Tamoxifen and Goserelin or Leuprolide	
			For post-menopausal women, endocrine therapy consists of Letrozole	
**NCT03294694**	I	Terminated	Ribociclib (Cyclin D1 and CDK4/6 inhibitor) combined with PDR001 (anti-PD1) with or without Fulvestrant (selective estrogen receptor degrader)	Metastatic HR+ breast cancer, HER2- breast cancer and metastatic epithelial ovarian cancer
**NCT03195699**	I	Active	TTI-101 (STAT3 inhibitor)	Breast, colorectal, hepatocellular, non-small cell lung cancer, head and neck squamous cell carcinoma, gastric adenocarcinoma and melanoma
**NCT05384119**	I/II	Recruiting	TTI-101 (STAT3 inhibitor) in combination with Aromatase inhibitor and Palbociclib (CDK4/6 inhibitor)	Breast cancer
**NCT05491226**	II	Not yet recruiting	Pembrolizumab (anti-PD1) in combination with radiation therapy and Axatilimab (anti-CSF-1R antibody)	TNBC
**NCT03448042**	I	Recruiting	Runimotamab (anti-HER2/CD3 bispecific antibody) in combination with Trastuzumab (anti-HER2 antibody) and Tocilizumab (anti-IL6 antibody)	Solid cancers

## Immune landscapes of breast cancer

The addition of immunotherapies such as those targeting programed death 1 (PD1) receptor on activated T-cells, programmed death ligand 1 (PDL1) and cytotoxic T-lymphocyte associated 4 (CTLA4) protein has become a standard of care for treatment of melanoma (Huang and Zappasodi, 2022). This is attributed to the highly immunogenic nature of melanoma allowing a permissive environment for immune based interventions (Huang and Zappasodi, 2022). More recently, the anti-PD1 antibody, Pembrolizumab, in combination with chemotherapy has been approved for the treatment of mismatch repair deficient colorectal tumors (Andre et al., 2020) and triple negative breast cancers (TNBCs) (Schmid et al., 2020). With the rise of immunotherapy to treat several solid tumors (Esfahani et al., 2020), a shift towards an immune-based rather than exclusively a histological classification of tumors may allow better segregation of responders and non-responders to the appropriate therapy. In TNBCs, RNA sequencing (seq.) suggested the existence of at least four subtypes within TNBCs including an immunomodulatory subtype, characterized by high T-cell infiltration and upregulation of immune checkpoint inhibitors (Jiang et al., 2019). These findings support that only a subset of TNBCs would respond to immune checkpoint blockade (ICB). Thus, it could be appreciated that classification of breast tumors using the traditional molecular and histological subtypes would not be sufficient to predict responders and non-responders within each breast cancer subtype.

An emerging concept has classified solid tumors into several immune categories. Immune hot (also referred to as immune inflamed), which are classified as being heavily infiltrated by T-cells and express markers of immune cell activation and exhaustion, are robust responders to immunotherapies (Galon and Bruni, 2019; Gruosso et al., 2019; Hammerl et al., 2021). On the other end of the spectrum are immune cold (also referred to as immune desert or ignored) tumors, which exhibit little to no T-cell infiltration and are unresponsive to immunotherapies (Galon and Bruni, 2019; Gruosso et al., 2019; Hammerl et al., 2021). Intermediate immune phenotypes (also referred to as immune altered) where immune cells are restricted to the tumor margin (marginally restricted) or restricted to the stroma (stromal restricted) add to the complexity of the immune microenvironment and are associated with minimal response to immunotherapy (Galon and Bruni, 2019; Gruosso et al., 2019; Hammerl et al., 2021). Moreover, intratumor heterogeneity may result in only a partial response to immunotherapy as well as allow for clonal selection for a resistant population. This is illustrated in the case of hypoxic tumors, with areas being of different immune status depending on their hypoxic status (Pietrobon and Marincola, 2021).

Recent studies using transcriptomic profiling of all subtypes of breast cancer divided tumors into three immune categroies based on immune signature scores: immune high, medium and low (Yao et al., 2021). Immune high tumors were defined as having the highest expression of PDL1 and CD8 to CD4 T-cell ratio (Yao et al., 2021). Interestingly, a higher proportion of TNBCs and HER2+ tumors were classified as immune high tumors whereas estrogen receptor (ER) and progesterone receptor (PR) positive tumors (the HR+ tumors) were mainly immune low (Yao et al., 2021). Analysis from The Cancer Genome Atlas (TCGA) supported this notion by demonstrating TNBCs and HER2+ breast cancers exhibit increased immune metagene expression compared to those of the ER+ subtype (Safonov et al., 2017). This work (Safonov et al., 2017; Yao et al., 2021) argues that ER/PR+ tumors are of lower immunogenicity and thus require tailoring therapeutic regimens to increase their response to immunotherapy.

An increase in number of T-cells infiltrating the tumor is associated with better patient outcome when treated with chemotherapy only (Denkert et al., 2018), chemotherapy with immunotherapies (Li et al., 2021a) as well as in combination with anti-HER2 targeted therapies, such as Trastuzumab (Loi et al., 2014). Therefore, it has become critical to understand the mechanism governing the different TIMEs, which may elucidate better drug targets which ultimately may improve patients clinical progression.

**FIGURE 2:**
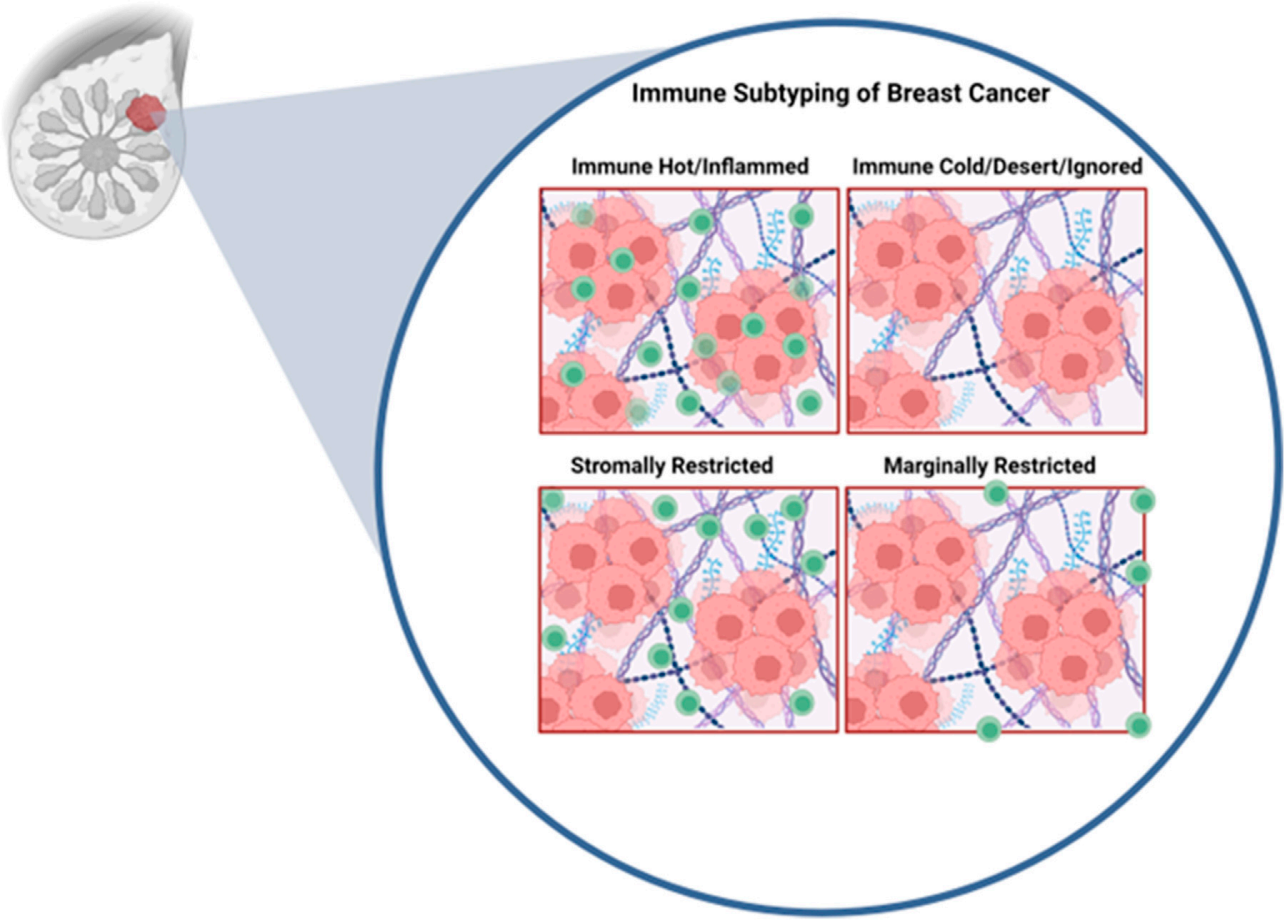
Immune profiles of breast cancer. Inspired by Grusso et al. In the immune hot subtype, cytotoxic T-cells are able to fully infiltrate the tumor. Immune cold tumors are fully devoid of cytotoxic T-cells. Stromally restricted tumors are infiltrated by T-cells only in the stroma but not tumor nest. Marginally rrestricted tumors have T-cells limited to the tumor margin and not capable to reaching the tumor nest. Figure generated with biorender.com.

In addition to absolute numbers of T-cells in the tumor being predictive of therapeutic response, studies have demonstrated that the spatial localization of immune cells might be critical in tumor classification and predicted response to therapy (Gruosso et al., 2019; Hammerl et al., 2021) Work by Gruosso et al. (Gruosso et al., 2019) on TNBCs clustered tumors into four subtypes based on the spatial localization of CD8^+^ (cytotoxic) T-cells (Gruosso et al., 2019) (Figure 2). Immune cold tumors were divided into two subcategories; immune dessert (ignored), which have no CD8^+^ T-cells cells in the tumor core or margins, and marginally restricted (or excluded), with CD8^+^ T-cells cells exclusively in the margins. Immune hot tumors were divided into stromal restricted (or excluded) with CD8^+^ T-cell only in the tumor associated stroma and fully inflamed which have CD8^+^ T-cells both in the stroma and the tumor nest. Profiling of TNBC tumors exhibiting inflamed, ignored, and excluded (in the margins or stroma) T-cell infiltration phenotypes demonstrated profound transcriptional changes and led to generation of gene signatures that are predictive of T-cell infiltration patterns (Hammerl et al., 2021). Moreover, it was demonstrated that immune inflamed tumors (those with a high immune inflamed gene signature) responded significantly better to anti-PD1 therapy assessed by better overall survival compared those of the immune ignored and excluded categories (Hammerl et al., 2021). These results suggest that transcriptomic profiling of tumors may allow for prediction of the immune landscape and thus response to immunotherapy, allowing therapies to be tailored accordingly. In metastatic HER2+ breast cancer, CD8^+^ T-cells reaching the tumor core, but not the invasive margin or the stroma, was associated with longer survival with metastasis (Honkanen et al., 2017). Overall, these findings argue that understanding the molecular programs that regulate T-cell spatial localization are critical for improving T-cell activity as well as improved response to immune based therapies.

There is a developing consensus that both the number and the spatial localization of T-cells (and potentially other immune cell types) are an important predictor of survival as well as response to approved immunotherapies in breast cancer, particularly in TNBCs (Hammerl et al., 2021). While we described at least three different spatial immune profiles; immune hot, immune cold and excluded (either margin or stroma), most of the studies described in this manuscript rely on the simplistic immune cold (low immune infiltrate in general) and immune hot (inactive/exhausted T-cells). Therefore, for the rest of this manuscript we will follow the nomenclature of the cited work. Additionally, most of this work has been focused on TNBCs. Further research is required to better categorize the TIME in HER2+ and ER+/PR+ tumors and thus improve the prospect of immune based therapies for these breast cancer subtypes, that account for the majority of cases (Harbeck et al., 2019).

## Approaches to treat immune cold tumors

Given that increasing numbers of tumor infiltrating CD8^+^ T-cells cells are associated with better prognosis and response to immunotherapy, work aimed at promoting T-cell infiltration into the tumor (core) could help improve patient outcomes as well as sensitize them to already approved immunotherapies.

### Induction of IFN expression through sustaining STING activation

Immune cold tumors arise due to inadequate induction of an innate immune response during early tumor progression (Bonaventura et al., 2019). This is usually due to reduced expression of interferons (IFNs) and interferon stimulated genes (ISGs) (Bonaventura et al., 2019; Galon and Bruni, 2019; Liu and Sun, 2021). Reduced expression of interferons leads to loss of major histocompatibility complex (MHC) class 1 expression which impedes recognition by T-cells (Dhatchinamoorthy et al., 2021). Loss of MHC class 1 is observed in about 60% of TNBCs which may account for the lack of response to immunotherapies in patients (Dusenbery et al., 2021). One of the key regulators of the interferon response is stimulator of interferon genes (STING) (Zhu et al., 2019; Decout et al., 2021). STING is a cytosolic sensor of the cyclic dinucleotide 2′3′-cyclic guanosine monophosphate–adenosine monophosphate (2′3′-cGAMP), which is produced by cyclic GMP-AMP synthase (cGAS) due to cytosolic DNA as a result of cell death or viral infection (Decout et al., 2021). STING activates tank-binding kinase 1 (TBK1), leading to activation of interferon response factor (IRF) three which acts as a transcription factor for ISGs (Decout et al., 2021). Induction of interferons act as a “red flag” for the immune system and results recruitment of anti-tumor immune cells. Blocking this cascade at any stage could result in an immune cold, interferon depleted tumor (Zhu et al., 2019). It is important to note, that 2′3′-cGAMP functions as an immune transmitter between different cancer cells as well as cancer cells and immune cells (such as dendritic cells) whereby one cell can activate STING in a neighbouring cell (Carozza et al., 2020) leading to a positive feedback cascade and increased inflammation. However, excessive inflammation, a hallmark of cancer (Hanahan and Weinberg, 2011), leads to disruption of tissue homeostasis and recruitment of inhibitory immune cells such as regulatory T-cells (T_regs_), myeloid derived suppressor cells (MDSCs), macrophages and upregulation of immune checkpoint molecules which sustains an immunosuppressed immune hot TIME.

As a mechanism of stimulating STING in immune cold tumors, either 2′3′-cGAMP or agonists (small molecules that activate STING) have been implemented to stimulate interferon expression and promote an anti-tumor immune response (Deng et al., 2014). Using the 4T1 TNBC model, the STING agonist, cyclic diguanylate, lead to induction of IFNβ expression which was coupled to an increase in activation of STING and its downstream effectors (Yin et al., 2022). Cyclic diguanylate synergized with atezolizumab (anti-PDL1) to reduce tumor growth through activation of cytotoxic T-cells and suppression of T_regs_ (Yin et al., 2022). Other work on a HER2+ model using the STING activator, ADU-S100, demonstrated that STING activation can induce tumor regression as well as protect against tumor rechallenge in non-tolerized animals (Foote et al., 2017). The effect of ADU-S100 was dependent on both CD8^+^ and CD4^+^ T-cells (Foote et al., 2017). Moreover, blockade of immune checkpoints, OX40 receptor and anti-PDL1 lead to a significant increase in ADU-S100 efficacy and complete regression of tumors (Foote et al., 2017).

Several STING agonists are currently in early phases of clinical trials as an adjuvant or in combination with other immunotherapies (NCT05070247, NCT04144140, NCT03956680, NCT03843359, NCT03010176, NCT03249792, NCT04096638). Additionally, already approved poly ADP ribose polymerase (PARP) inhibitor, Olaparib, in a TNBC mouse model demonstrated its capacity to induce CD8^+^ T-cell infiltration *via* activation of STING (Pantelidou et al., 2019) as well as promote upregulation of PDL1 in several human derived cell lines (Jiao et al., 2017) suggesting that inducing DNA damage may promote a strong anti-tumor immune response through STING activation. Noteworthy, that increasing the tumor mutational load is associated with favourable immune infiltration and response to immunotherapies (Strickler et al., 2021), potentially due to elevated basal activation of STING. However, only a very small fraction of breast cancers have a high mutational load compared to immunogenic tumors such as lung and melanoma (Thomas et al., 2018; Barroso-Sousa et al., 2020). Additionally. HR + tumors harbour a smaller proportion of tumors with a high mutational burden (Thomas et al., 2018). Mechanisms that increase the mutational load might be clinically beneficial, especially for HR + breast cancer. Indeed, inactivation of the DNA mismatch repair (MMR) by targeting MutL homologue 1 (MLH1) leads to an increased mutational load due to reduced genomic stability, triggering an immune response that sensitizes tumors to anti-PD1 (Germano et al., 2017).

In a cDNA library screen, HER2, which is highly expressed in ∼25% of breast cancers (Harbeck et al., 2019), was found to impede STING activation (Wu et al., 2019). Detailed analysis of this phenomenon demonstrated that the intracellular domain (ICD) of HER2 physically sequesters STING, preventing interaction with TBK1/IRF3 (Wu et al., 2019). Additionally, AKT1 activation downstream HER2, phosphorylates TBK1 at serine 510 which inhibits its downstream activity and association with STING (Wu et al., 2019). This data strongly argues that oncogene mediated signaling can impact the innate immune response and result in an immune cold TIME, which is amenable to receptor tyrosine kinase inhibitors (RTKi) such as lapatinib (Wu et al., 2019). Interestingly, activation of DNA sensing increased the HER2-AKT1 cascade (Wu et al., 2019). We envision that this may behave as a mechanism of resistance in tumors treated with STING activators.

Novel approaches at regulating STING have led to development of small molecules targeting ecto-nucleotide pyrophosphatase/phosphodiesterase 1 (ENPP1) which is an endoplasmic reticulum and cell surface enzyme that degrades natural STING ligand 2′3′-cGAMP as well as ATP/GTP to AMP and GMP (Li et al., 2014; Carozza et al., 2022; Gangar et al., 2022; Goswami et al., 2022). Inhibition of ENPP1 using a small molecule inhibitor was sufficient to induce an active immune response which synergized with radiation therapy (Carozza et al., 2020) potentially due to reduced degradation of 2′3′-cGAMP produced by irradiated cells. Moreover, other studies demonstrated that chromosomally unstable breast tumors suppress an immune response against them *via* selective upregulation of ENPP1 which degrades 2′3′-cGAMP that may be produced in response to dsDNA released into the cytosol following nuclear envelope ruptures (Li et al., 2021b). Indeed, knockout of ENPP1 in TNBC syngeneic models reduced metastasis to the lung and led to a massive increase in immune cell infiltration at the primary tumor (Li et al., 2021b). Moreover, knockout of ENPP1 synergized with PD1 blockade (Li et al., 2021b). Both patient tumors and mouse models demonstrated that ENPP1 is elevated in relapsed tumors of the TNBC and HER2+ subtypes (Ruiz-Fernandez de Cordoba et al., 2022). ENPP1 promoted neutrophil extracellular trap (NET) formation *via* tumor cell expression of haptoglobin, supporting relapse and protecting the tumor from radiotherapy (Ruiz-Fernandez de Cordoba et al., 2022). Additionally, both expression of interferons and STING activation are associated with differentiation of the tumor, reduced stem cell capacity and reduced expression of epithelial-mesenchymal transition (EMT) gene signature (Cheng et al., 2020; Cheon et al., 2022; Goswami et al., 2022). Targeting ENPP1 reduced expression of EMT markers and signatures (Goswami et al., 2022). Only one documented case of first-in-human ENPP1 inhibitor in addition to anti-PD1 was performed (Csiki et al., 2022), but no large scale trials have been preformed thus far. We posit that inhibition of ENPP1 may provide strong clinical benefit especially to immune cold TNBCs and HER2+ breast cancers. Combination of 2′3′-cGAMP (Ohkuri et al., 2017) or radiation (Carozza et al., 2020) coupled to ENPP1 inhibition may demonstrate a synergistic effect due to the prolonged accumulation of 2′3′-cGAMP in the extracellular milieu.

AMP produced by ENPP1 is subjected to further hydrolysis by CD73 (Allard et al., 2020). The resultant adenosine may then bind adenosine receptors found on stromal or cancer cells to reinforce an immunosuppressive microenvironment (Allard et al., 2020). CD73 expression was found to be highest in TNBC compared to other breast cancer subtypes and was significantly associated with worse prognosis (Loi et al., 2013). Activity of CD73 in TNBC induced immunosuppression and promoted resistance to anthracyclines (Loi et al., 2013). Targeting CD73 using a monoclonal antibody increased the number of tumor specific CD8^+^ T-cells and induced INFγ expression (Loi et al., 2013). Combination of anthracycline (doxorubicin) with anti-CD73 delayed death due to metastasis of animals post surgical resection of their primary tumor (Loi et al., 2013). Moreover, anti-CD73 synergized with both anti-PD1 and anti-CTLA4 (Loi et al., 2013). Expression of CD73 has also been correlated with resistance to anti-HER2 targeted therapy, Trastuzumab (Turcotte et al., 2017). Neutralization of CD73 synergized with anti-HER2 monoclonal therapy, eliminated HER2+ tumor growth in syngeneic models as well as reduced pulmonary metastasis (Turcotte et al., 2017). Anti-CD73 targeting antibodies as well as CD73 inhibitors are currently in early phase clinical trials in combination with immune checkpoint blockades and Trastuzumab (NCT03616886, NCT03875573, NCT03454451, NCT05431270, NCT05143970, NCT05001347, NCT04148937).

### Use of chemotherapy and radiation

Chemotherapy in combination with anti-PD1 is approved for PDL1+ TNBC (Cortes et al., 2022). While the idea of eventually phasing out chemotherapy and radiation is highly tempting due to their systemic adverse effects; they still remain viable options to prime an immune response and potentially induce upregulation PDL1 and other co-inhibitory molecules (Zitvogel et al., 2008).

DNA damage following radiation induces STING activation which promotes recruitment of dendritic cells which act as antigen presenting cells priming T-cell mediated immunity (Dhatchinamoorthy et al., 2021) (impact is under clinical assessment NCT03978663). Both chemotherapy and radiation can induce cell and DNA damage, generating neoantigens and inducing antigenicity which may sensitize tumors to immunotherapies (Zitvogel et al., 2008; McGranahan et al., 2016; Galon and Bruni, 2019). Additionally, chemotherapy has been demonstrated to selectively deplete MDSCs, T_regs_ and restores T- and natural (NK) cell functions (Ghiringhelli et al., 2007; Vincent et al., 2010). DNA damage following radiation induces STING activation which promotes recruitment of dendritic cells that act as antigen presenting cells to prime T-cell mediated immunity (Deng et al., 2014). Clinical studies (NCT03978663) are currently underway to correlate radiation treatment with immune cell recruitment and patient outcome.

Resistance to radiation and chemotherapy posses an additional challenge, as tumors need to respond to radiation or chemotherapy to effectively prime an immune response. Resistance to radiation has been linked to hypoxia (briefly discussed in the next section) where lack of molecular oxygen prevents generation of free radicals to react with DNA (Wang et al., 2021). Strategies aimed at promoting tumor oxygenation by vasculature normalization have demonstrated the capacity to sensitize tumors to radiation and improve chemotherapy delivery in multiple tumor models (Telarovic et al., 2021). Tyrosine kinase inhibitor Sunitinib is under clinical investigation in combination with chemotherapy to induce vasculature normalization to enhance chemotherapy delivery (NCT01803503). Blood vessel normalization and perfusion is driven by IFNγ expressing cytotoxic T-cells and is a predictor of response to ICB, anti-PD1 and anti-CTLA4, and the anti-HER2 monoclonal, Trastuzumab (Baker et al., 2018; Zheng et al., 2018). Therefore, strategies at improving vasculature perfusion may permit increased cytotoxic T-cell infiltration, which establishes a positive feedback loop, increasing vasculature perfusion further allowing better delivery of chemotherapy and anti-tumor immune cells. Thus, improved vasculature perfusion may also allow better tumor oxygenation and improved immune responses.

### Targeting hypoxia to promote immune cell trafficking

Hypoxia is regarded as a critical phenomenon in breast cancer (Zhang et al., 2021). Indeed, an abundance of work has demonstrated that hypoxia and upregulation of hypoxia inducible factor 1α (HIF1α) in the epithelium regulates breast cancer progression and metastasis *via* promoting angiogenesis, induction of cancer stem phenotypes and EMT (Muz et al., 2015). Additionally, hypoxia promotes immune suppression either by inhibiting T-cell activity or through exclusion of immune cells by a barrier comprised of low pH, low glucose, and low oxygen conditions (Wang et al., 2021; Zhang et al., 2021). Interestingly, CD73 is under the control of hypoxia and potentially contributes to hypoxia mediated immunosuppression (Allard et al., 2020). Spatial analysis of 4T1 tumors demonstrated that hypoxic areas (HIF1α+) are poorly infiltrated with T-cells as expected (Ma et al., 2022). This work identified that through HIF1α, hypoxia induced silencing of INFγ and tumor necrosis factor (TNF) through histone deacetylase (HDAC) and enhancer of zeste homolog 2 (EZH2) mediated epigenetic silencing in T-cells (Ma et al., 2022). Inhibition or genetic ablation of HIF1α, HDAC or EZH2 sensitized anti-PD1 resistant 4T1 tumors to anti-PD1 (Ma et al., 2022). Knockout of HIF1α in the macrophages of polyoma middle T (PyMT) tumors increased proliferation and cytotoxic activity of T-cells, emphasizing that hypoxia may promote immunosuppression through macrophage function as well (Doedens et al., 2010).

While low oxygen stabilizes HIFs and regulates a hypoxia induced response, receptor tyrosine kinases (RTKs) such as the epidermal growth factor (EGFR) family, which are commonly highly expressed in breast cancers can stabilize HIFs (Lauzier et al., 2007; Lee et al., 2009; Nilsson et al., 2010; Turpin et al., 2016). Interestingly, it was demonstrated that through HIF2α, hypoxia can promote translation of *EGFR* mRNA (Franovic et al., 2007; Uniacke et al., 2012) implying the presence of a positive feedback loop between HIFs and EGFR. Knockout of AXL RTK in a HER2+ driven mouse model reduced expression of hypoxic markers, reactivated the immune response and improved vasculature perfusion in the primary tumors leading to strong reduction in lung metastasis (Goyette et al., 2021). Inhibition of AXL using R428 synergized with PD1 blockade at blocking metastasis (Goyette et al., 2021). Clinical trials assessing the AXL inhibitor, Bemcentinib, with Pembrolizumab (anti-PD1) on TNBC was attempted on a small cohort of patients (NCT03184558) but none achieved complete response leading to termination of the trial. Newer trials are currently underway to assess impact of chimeric antigen receptor (CAR) T-cells against AXL as well as other RTKs including HER2 and EGFR on advanced tumors (NCT04842812).

### Targeting EZH2 to induce inflammatory cytokine expression

EZH2 acts as the catalytic subunit of polycomb repressive complex 2 (PRC2) and places a repressive methyl mark on lysine 27 of histone 3, H3K27me3 (van Mierlo et al., 2019). Progression of PyMT-driven tumors is correlated with an increase in H3K27me3 repressive marks (Cai et al., 2018). EZH2 knockout in PyMT and HER2-driven models demonstrated profound impact on tumor initiation, progression, and metastasis, supporting that EZH2 behaves as on oncogene by suppressing expression of tumor suppressors (Hirukawa et al., 2018; Smith et al., 2019). We have previously demonstrated that inhibition of EZH2 in a HER2+ mouse model induces tumor cell expression of endogenous retroviral elements that promote expression of IFNs and sensitize tumors to anti-HER2 targeted therapy (Hirukawa et al., 2019). Work using ovarian cancer models demonstrated that inhibition of EZH2 induced expression of inflammatory cytokines C-X-C ligand (CXCL) 9 and CXCL10 that promoted recruitment of cytotoxic T-cells into the tumor (Peng et al., 2015). Studies from other tumor models have demonstrated that EZH2 inhibits expression of PDL1 (Xiao et al., 2019) and the MHC class 1 antigen processing pathway (Burr et al., 2019). These studies support that epigenetic inhibition may impact induction of a robust immune response both through the tumor cell proper as well as through direct action on immune cells. The EZH2 inhibitor, SHR2554 is currently in clinical trials in combination with PARP, androgen receptor and cyclin dependent kinases four and 6 (CDK4/6) inhibitors (NCT04355858 and NCT03805399).

### Oncolytic viruses to stimulate the immune response

Oncolytic viruses (OVs) are naturally occurring or genetically modified viruses that are capable of selectively infecting and destroying tumor cells (Jin et al., 2021). OVs induce tumor cell death, resulting in release of pathogen-associated molecular patterns (PAMPs) and danger-associated molecular patterns (DAMPs) that can stimulate the anti-tumor immune response (Jin et al., 2021). While OVs are not currently approved, several are currently under pre-clinical and clinical evaluation (Carter et al., 2021; Jin et al., 2021). A herpes simplex virus (HSV)-based OV designated G47Δ, reduced pulmonary metastatic growth (Wang et al., 2012). Addition of IL-12 coding sequence to G47Δ (designated G47Δ-mIL12) killed both murine and human breast cancer cells *in vitro* and in syngeneic models. It also led to increased infiltration of cytotoxic T-cells and decreased MDSCs infiltration into the tumors (Ghouse et al., 2020). Several OVs are currently in clinical assessment in combination with other immunotherapies such as anti-PDL1 and CAR-T cells (NCT05600582, NCT05081492, NCT03004183, NCT04445844, NCT02779855, NCT04185311, NCT05076760, NCT04215146, NCT03740256, NCT05180851, NCT04521764).

### Introduction of genetically engineered anti-tumor immune cells

Given that T-cells are known for their ability to recognize and kill cancer cells, efforts at expanding them *ex vivo* and reintroducing them back into the patient in a process known as adoptive cell transfer (ACT), has been successful in highly immunogenic tumors (Luo et al., 2022). Recently, ACT of T-cells targeting mutant proteins (SLC3A2, KIAA0368, CADPS2 and CTSB) detected in a patient tumor in combination with interleukin two have been demonstrated to induce tumor regression (Zacharakis et al., 2018). Clinical investigations aimed at expanding anti-HER2 specific T-cells for reintroduction into patients in combination with Trastuzumab (NCT05378464) are currently actively recruiting. Moreover, genetically engineered CAR-T cells have been developed targeting commonly expressed breast cancer antigens such as HER2 (Szoor et al., 2020), EGFR (Liu et al., 2019), EpCAM (Osta et al., 2004), AXL (Zhao et al., 2020a) and c-MET (Tchou et al., 2017). Unfortunately, CAR-T cells are met with the same challenges as regular T-cells where the tumor microenvironment may not be permissive for their survival or activity, presence of inhibitory immune cells as well as lack of homing cues (Li et al., 2020). Studies have addressed this by stimulating STING using 2′3′-cGAMP or DMXAA which improved trafficking and CAR-T cells targeting HER2 in the TIME of breast cancer (Xu et al., 2021). Tumor heterogeneity, whereby some tumor cells may lack the antigen targeted by the CAR or loss of MHC class 1 which is required for recognition of the tumor cell may hinder the impact of CAR-T cells (Li et al., 2020; Luo et al., 2022).

Development of CAR-natural killer (CAR-NK) cells targeting breast cancer tumor antigens (Liu et al., 2020; Portillo et al., 2021) harbours several advantages over CAR-T cells, importantly not requiring MHC matching (Khawar and Sun, 2021). Therefore, CAR-NK need not be from the same patient, have a low risk of graft vs. host disease and do not require MHC to be expressed on the tumor cell (Khawar and Sun, 2021). Additionally, CAR-NKs demonstrate enhanced tumor killing potential compared to CAR-T cells coupled with reduced risk of cytokine release syndrome, which is acute systemic inflammation that could be life threatening (Shimabukuro-Vornhagen et al., 2018; Khawar and Sun, 2021; Portillo et al., 2021).

CAR-macrophages (CAR-Ms) have similar advantages to CAR-NKs with the addition of macrophages having better capacity at infiltrating the tumor (Klichinsky et al., 2020). CAR-M targeting HER2, decreased tumor burden and increased overall survival of tumor bearing mice (Klichinsky et al., 2020). Moreover, they induced expression of inflammatory cytokines capable of converting tumor resident M2 macrophages to the M1 phenotype and boosted anti-tumor T-cell activity (Klichinsky et al., 2020). To our knowledge, no clinical trials are currently underway assessing CAR-NKs or CAR-Ms in breast cancer.

**FIGURE 3:**
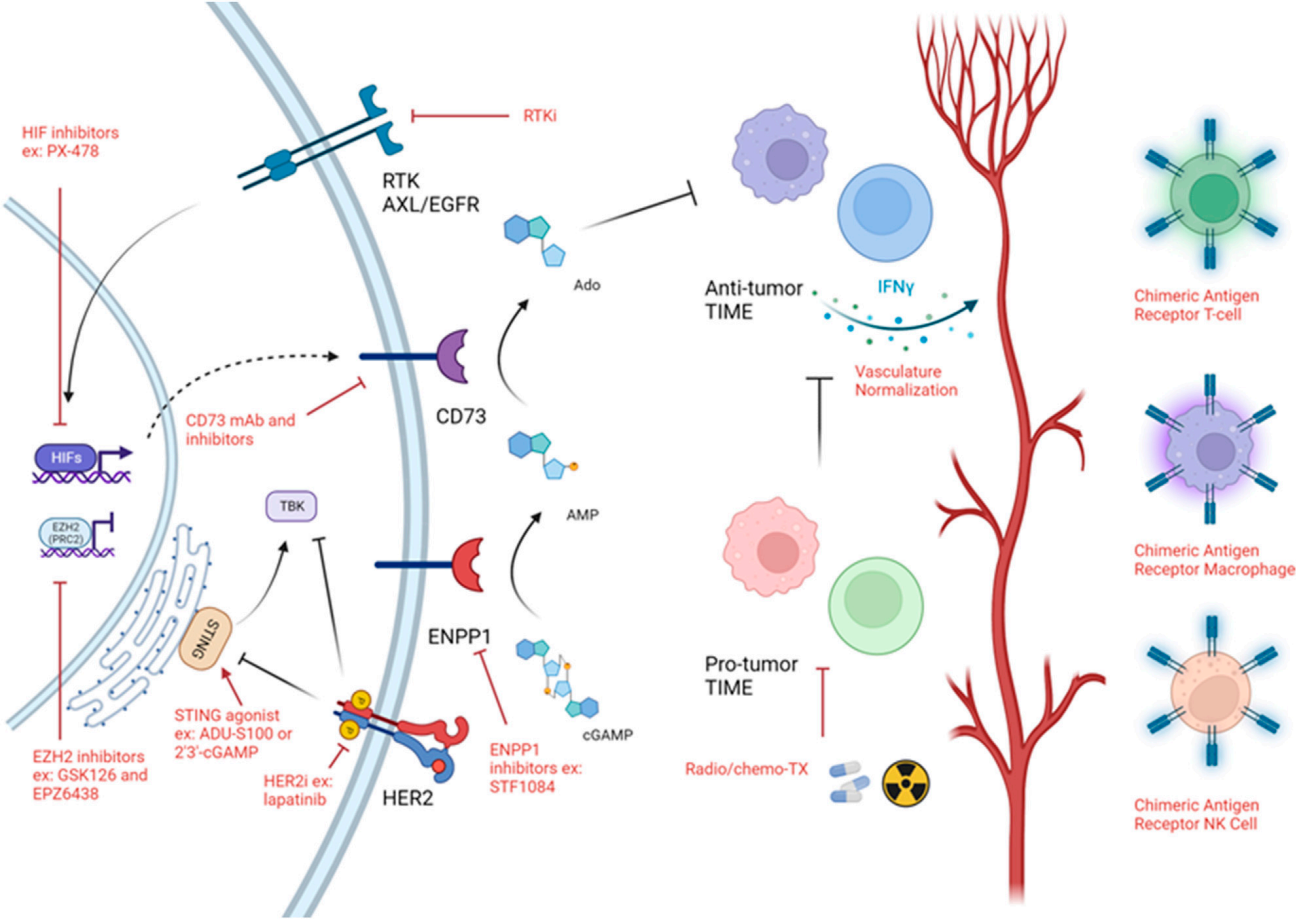
Strategies targeting immune cold TIME. Schematic of strategies used to target immune cold timors. Black arrows demonstrates a biologically activating pathway. Black flat head demonstrates a biologically inhibitory pathway. Red arrow implies activating intervention. Red flat head implies inhibitory intervention. Figure generated with biorender.com.

Overall, while immune cold tumors represent a difficult to treat tumor and are associated with poor patient outcome, there is encouraging data suggesting that reactivation of the tumor immunity cycle *via* STING agonists or targeting ENPP1 or CD73 is sufficient to increase immune cell infiltration into the tumor especially in combination with either chemotherapy or radiotherapy. Targeting hypoxia both directly (through HIF inhibition) and indirectly (through RTKs) may relieve T-cell exclusion therefore, sensitizing patients to traditional therapies and immunotherapies. Epigenetic rewiring of the tumor as well as the immune cells themselves may increase their activation and infiltration into the tumor. Oncolytic virus-based therapies may induce hotness in the tumor due to DAMP and PAMP generation. Finally, to increase the number of anti-tumor immune cells at the tumor generation of genetically engineered T-cells, NKs and macrophages may help reshape the TIME (Figure 3).

## Targeting hormone receptor positive breast cancer—An immune cold subtype

As evident in this review [as well as in others (Goldberg et al., 2021)], significant effort has been focused on understanding the TIME of TNBCs and to a lower extent HER2+ breast cancer given that they represent the most aggressive subtypes. However, 70% of breast cancer diagnoses are ER/PR+ (collectively referred to as hormone receptor positive, HR+), thus understanding the TIME of these tumors is of clinical importance (Goldberg et al., 2021). While endocrine therapy is usually restricted to HR+ breast cancer, the ER is expressed in other cell types, including immune cells (Chakraborty et al., 2023). Moreover, in premenopausal women, significant levels of estrogen may reach the mammary gland and reshape the TIME (Svoronos et al., 2017).

Efforts at understanding the TIME in HR+ breast cancers have been made through use of human breast cancer biopsies and clinical trials. It is now widely accepted that HR+ (particularly HR+/HER2-) status represents immune cold tumors that exhibit reduced T-cell infiltrate (Goldberg et al., 2021). Aforementioned, HR+ breast tumors harbour a lower tumor mutational burden (Thomas et al., 2018; Barroso-Sousa et al., 2020) coupled to significantly lower expression levels of MHC class I and PDL1 compared to other breast cancer subtypes (Zhang et al., 2017; Onkar et al., 2023). Consistent with this notion, activity of the ER is negatively correlated with IFN expression while PR signalling dampens the (signalling transducer and activator of transcription 1 (STAT1)-IFN signaling axis (Lee et al., 2016; Goodman et al., 2019). Additionally, increasing number of stromal T-cells in HR+ breast cancer is a negative prognostic factor (Denkert et al., 2018) and having lower T-cell counts after chemotherapy is associated with improved relapse free survival in HR+ disease (Watanabe et al., 2018). Whether the lower stromal T-cell count is due to increased infiltration into the tumor nest remains to be fully addressed and would explain these discrepancies. Overall, it has become abundantly clear that HR+ tumors are fortified with multiple mechanisms that allow them to support an immune cold TIME.

Estrogen deprivation through inhibition of aromatase by letrozole reduced levels of FOXP3+ T_regs_ (Generali et al., 2009) with a prominent increase in CD8^+^/T_regs_ ratio especially in responders (Chan et al., 2012). Additionally, progesterone supplementation was found to decrease natural killer cell and natural killer T-cell population in a mouse model of PR+ disease (Werner et al., 2021), suggesting that both female hormones may act in concert to generate an immunosuppressive TIME. Tamoxifen is one of the most prescribed breast cancer medications in the adjuvant setting. Studies have demonstrated that tamoxifen treatment induced proliferation of active T-cells (Komi and Lassila, 1998) and increased natural killer cell activity (Richards et al., 2016). Ablation of estrogen activity reduced recruitment of myeloid derived suppressor cells and boosted T-cell activity through its action on the JAK/SRC/STAT3 pathway in the immune cells (Svoronos et al., 2017) indicating that anti-estrogens may have implications in non-ER+ cancers as well. Indeed, estrogen has been demonstrated to induce pro-tumor M2 macrophage polarization (Toniolo et al., 2015) as well as inhibit production of IFNγ and increased expression of indoleamine 2,3-dioxygenase (IDO) which is a T-cell inhibitor, by mature dendritic cells (Liu et al., 2002; Xiao et al., 2004). Attempts have further been made to bolster the anti-tumor immune response generated by tamoxifen treatment through combination with IFNβ and IL2, which prolonged survival of patients harbouring distant metastasis (Nicolini and Carpi, 2005; Nicolini et al., 2005). However, in autoimmune encephalomyelitis, tamoxifen induced upregulation of Th2 cytokine expression (Bebo et al., 2009), as well as *in vitro* neutrophil NET formation (Corriden et al., 2015). Prolonged treatment of tumors with ER antagonist, fulvestrant, induced upregulation of PDL1 that contributed to immune exhaustion (Huhn et al., 2022). In summary, while tamoxifen and biosimilar agents modulate the TIME positively by increasing immune infiltrate, new accumulating evidence suggests that prolonged treatment with such molecules may establish an immunosuppressive microenvironment. While this immunosuppressive microenvironment may allow for immune escape, it is highly amenable to immunotherapy, further supporting the idea of combination approach.

Activating mutations in the gene encoding the ER (*ESR1*) have been identified in the context of metastatic breast cancer previously treated with tamoxifen and aromatase inhibitors (Turner et al., 2020; Zundelevich et al., 2020). Mutations in the ER are associated with reduced progression free survival and overall survival (Turner et al., 2020; Zundelevich et al., 2020) as well as resistance to the HR+ breast cancer standard of care (Herzog and Fuqua, 2021; Liang et al., 2022). Interestingly, it was recently demonstrated that tumors harbouring mutations in the ER had elevated levels of inflammatory cytokines S100A8 and S100A9, T_regs_ and PDL1 positive macrophages in addition to expression of the immunosuppressive cytokine, chitinase-3-like 1 (CHI3L1) (Zhao et al., 2020b; Williams et al., 2021; Li et al., 2022). Neutralizing antibodies against CHI3L1 have demonstrated strong ability to revert immunosuppression and synergized with ICB in other cancer models (Ma et al., 2021) suggesting that targeting CHI3L1in ER mutant breast cancer may be a viable option to alleviate immunosuppression that resulted due to excessive inflammation. Overall, this data contends that while ER mutations promote therapeutic resistance to the traditional standard of care, the switch to an immune hot microenvironment may be associated with immune vulnerabilities. Several clinical trials assessing next-generation, selective estrogen receptor modulators or degraders are currently in progress to identify ones that have a positive clinical impact on patients harbouring mutant ER tumors/metastases [summarized in (Herzog and Fuqua, 2021)].

CDK4/6 inhibition in combination with aromatase inhibitors or ER modulators (such as letrozole and fulvestrant) is currently emerging as the standard of care for HR+ breast cancers due to improved patient survival in clinical trials (Im et al., 2019; Slamon et al., 2020). Emerging clinical and preclinical evidence has suggested that CDK4/6 inhibitors relieve immune suppression (Goel S et al., 2017; Deng et al., 2018; Scirocchi et al., 2022) through increased antigen presentation, expression of endogenous retroviral elements and induction of a Type III IFN response (Goel S et al., 2017). This has warranted the need for clinical trials to address the impact of combined CDK4/6 inhibition, ER modulation and immunotherapy such as anti-PD1 or anti-PDL1 (NCT02778685, NCT03573648 and NCT03294694) (Rampioni Vinciguerra et al., 2022).

Overall, the TIME of HR+ breast cancer is highly complex. Additionally, understanding the TIME to the same degree as TNBC and HER2+ breast cancer is needed to better prognosticate and improve treatment of the various TIME of HR + breast cancer that potentially exists.

## Targeting immune hot tumors and metastasis by resolving inflammation

Chronic inflammation is a hallmark of cancer (Hanahan and Weinberg, 2011). Indeed, several benign pathologies marked with chronic inflammation, such as liver cirrhosis or inflammatory bowel disease, are associated with an increased risk of developing cancer (Sangiovanni et al., 2004; Grivennikov et al., 2010; Beaugerie et al., 2013). Oncogenesis is promoted by an unresolved inflammatory response which leads to the accumulation of stromal cells and loss of tissue homeostasis (Hanahan and Weinberg, 2011; Quail and Joyce, 2013). Excessive inflammation and expression of cytokines such as IFNs induce expression of immune checkpoint molecules such as PDL1, which in turn negatively regulate anti-tumor immune cells, leading to immunosuppression that is essential for tumor progression (Hanahan and Weinberg, 2011; Galon and Bruni, 2019). Therefore, strategies treating such tumors attempt to resolve excessive inflammation while making efficient use of immunosuppressive molecule inhibition to counteract tumor-induced T-cell dysfunction.

### Tumor-intrinsic immunosuppression through oncogenic pathway induction

Activation of the nuclear factor kappaB (NFΚB) inflammatory pathway is observed predominantly in ER negative tumors through activation of RTKs and/or cellular stress (Biswas et al., 2004; Frasor et al., 2015; Wang et al., 2015). Moreover, active NFKB is correlated with larger, more advanced tumors and correlates with lung and brain metastasis (Wang et al., 2015). NFKB activity is associated with expression of inflammatory cytokines that promote Th2 polarization and maintaining a cancer stem cell population that facilitates oncogenesis (Liu et al., 2010; Wang et al., 2015; Zhang et al., 2022). Inhibition of NFKB delayed tumor onset and abrogated tumor progression in a PyMT driven model of breast cancer (Connelly et al., 2011). Recent work demonstrated that the reticuloendotheliosis viral oncogene homolog A (RELA) subunit of NFKB recruits cat eye syndrome chromosome region candidate 2 (CECR2), an epigenetic factor that induces gene expression of several inflammatory cytokines including colony stimulating factor 1 (CSF1) (Zhang et al., 2022). This interaction was important in recruitment of macrophages as well as their polarization to the M2 state in the lung metastatic niche (Zhang et al., 2022). Targeting CECR2 in mice harbouring orthotopically transplanted 4T1 cells reduced metastasis through reduced M2 macrophage levels and concomitant increase in cytotoxic T-cell activity (Zhang et al., 2022).

NFKB components have been shown to interact with STAT3 and coregulate inflammatory oncogenic pathways in breast cancer (Fan et al., 2013). In addition to STAT3’s ability to regulate the immune microenvironment, STAT3 transcriptional targets regulate anti-apoptotic pathways, EMT and expression of metalloproteinases (MPPs) that act in concert to regulate growth and metastasis (Ma et al., 2020). Interestingly, mammary specific knockout of STAT3 in the PyMT model did not impact nascent tumors, but they were eventually eliminated by an active innate and adaptive immune response (Jones et al., 2016). This finding contends that targeting STAT3 or one of its essential transcriptional targets may be sufficient to induce a robust anti-tumor immune response that leads to complete clearance of tumors. It is noteworthy that our STAT3 knockout was restricted to the mammary epithelium (Jones et al., 2016), future studies using STAT3 inhibitors (Zou et al., 2020) to address the role of STAT3 in other cell types of the TIME are essential for understanding the full role of STAT3 in immunosuppression. To this end, STAT3 activation (assessed using expression of phosphorylated STAT3) in astrocytes of the brain microenvironment supported invasive growth of breast-to-brain metastasis in part through reduced activation of T-cell activity and upregulation of CHI3L1 expression (Priego et al., 2018; Dankner et al., 2022). TTI-101, a STAT3 inhibitor, is currently in early phase clinical trials (NCT03195699 and NCT05384119). Of note, active STAT3 downstream interleukin (IL) six was demonstrated to bind estrogen response elements and promote gene expression independent of the ER or its coactivator forkhead box A1 (FOXA1) promoting metastasis and resistance to anti-estrogen-based therapy (Siersbaek et al., 2020).

### Targeting macrophages

**FIGURE 4:**
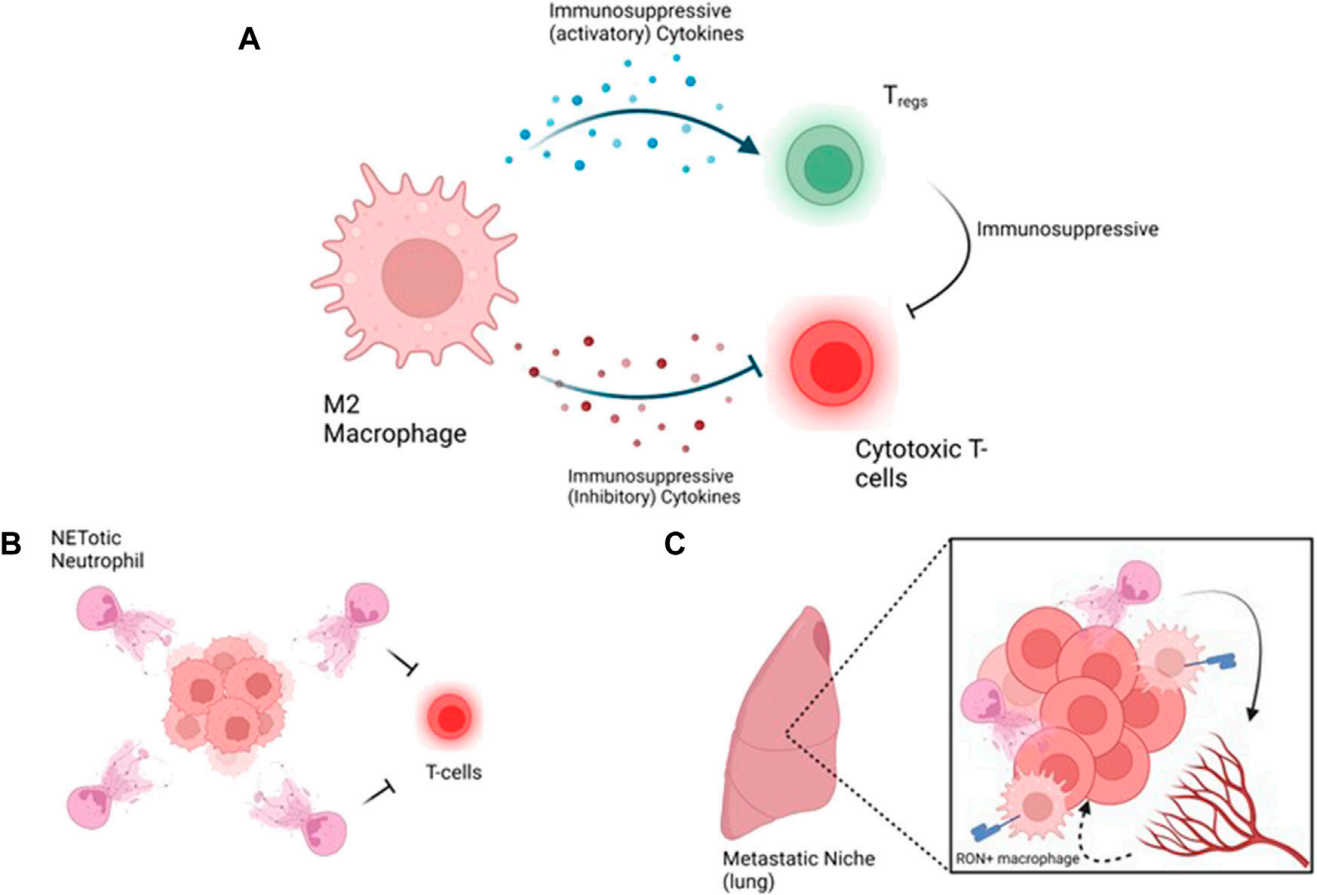
Strategies targeting immune hot tumors and metastasis, **(A)** A loop between macrophages promotes immunosuppression by activating inhibitory T-cells such as Tregs and inhibition of cytotoxic T-cells. Inhibitory T-cells emphasize the suppression of cytotoxic T-cells, **(B)** At the primary tumor site, NETotic neutrophils protect the tumors from cytotoxic T-cells, **(C)** At the metastatic site, neutrophils promote vascular permeability to allow extravasyion of the tumor cells and macrophages support the immunosuppressive environment *via* MSP/RON. Figure generated with biorender.com.

Macrophages are the most abundant immune cell type in the TIME, thus representing an attractive therapeutic target. Work from the PyMT mouse model demonstrated that as the tumor progresses, there is a decrease in mammary resident macrophages and an increase in tumor associated macrophages (Attalla et al., 2021). Macrophages are recruited to the tumor site due to expression of inflammatory cytokines, especially CSF1 and chemokine ligand (CCL) 2 (Lin et al., 2001; Franklin et al., 2014; Linde et al., 2018; Neubert et al., 2018). The importance of CSF1 was demonstrated using CSF1 knockout PyMT mice which exhibited delayed disease progression as well as prevented pulmonary metastasis through loss of vascular endothelial growth factor (VEGF) and epidermal growth factor (EGF) expression (Lin et al., 2001). Moreover, macrophages promote tumor cell migration and metastatic dissemination through secretion of EGF and Wnt1 (Wyckoff et al., 2007; Linde et al., 2018). Macrophages, especially those M2 polarized, establish the immunosuppressive microenvironment in immune hot subtypes through expression of cytokines that promote Th2 T-helper cell polarization and inhibition of cytotoxic T-cell function (Liu et al., 2021) (Figure 4A). Moreover, macrophages have been demonstrated to expresses PDL1 to promote exhaustion of T-cells (Ahmed et al., 2020). Inhibition of CSF1 receptor (CSFR) by small molecule (BLZ945) has demonstrated anti-tumor effects in glioma and breast cancer models through loss of M2 polarized macrophages and enhancement of T-cell recruitment to the tumor (Pyonteck et al., 2013; Strachan et al., 2013). Phase II clinical trial for TNBC combining Pembrolizumab (anti-PD1), radiation and Axatilimab (anti-CSFR) is currently ongoing (NCT05491226).

Another macrophage marker that has demonstrated pre-clinical interest is the “do not eat me” signaling axis consisting of signal regulatory protein-alpha (SIRPα) expressed on the macrophage (and other phagocytes) and CD47 expressed on cancer cells (Chen et al., 2022). CD47 is transcriptionally regulated by inflammation, though NFKB and TNF (Betancur et al., 2017) as well as hypoxia, through HIF1α (Zhang et al., 2015). CD47 has also been demonstrated to be an essential breast cancer stem cell marker (Zhang et al., 2015; Kaur et al., 2016; Chen et al., 2022). Engagement of CD47 with SIRPα inhibits the phagocytic capacity of macrophages (Chen et al., 2022). Antibody neutralization of CD47, induced a shift towards M1 macrophages and enhanced antigen presentation (Latour et al., 2001; Zhang et al., 2016; Chen et al., 2022). CD47 blockade also synergized with trastuzumab through enhancing antibody dependent cellular phagocytosis (Tsao et al., 2019). Multiple anti-CD47 antibodies have been developed and are under clinical assessment (Chen et al., 2022).

### Targeting neutrophils

Neutrophils are the most common cell type in human blood and have recently gained significant research interest. The neutrophil to lymphocyte ratio in many solid tumors is correlated with patient survival with a higher ratio being associated with worse patient survival (Bartlett et al., 2020; Gago-Dominguez et al., 2020). Neutrophils are recruited to the tumor (site of injury) through secretion of inflammatory cytokines such as tumor growth factor β (TGFβ), CXCL8 (also known as IL-8), CXCL1, CXCL2 as well as IFNs (SenGupta et al., 2021). Furthermore, neutrophils secrete MMP9 (SenGupta et al., 2021) a prominent enzyme that degrades the extracellular matrix, promoting cancer progression and tumor cell dissemination (Owyong et al., 2019). Moreover, expression of chemokine ligand CCL20 by the tumor cell induced expression of PDL1 on neutrophils which reinforces an immunosuppressive microenvironment *via* exhaustion of T-cells (Kwantwi et al., 2021).

Strikingly, neutrophils have been demonstrated to undergo a process known as NETosis, during which a neutrophil secretes its DNA into a weblike structure (Vorobjeva and Chernyak, 2020). NET formation is promoted by cancer derived factors such as granulocyte-CSF (G-CSF), CXCL5, CXCL6 (Ronchetti et al., 2021). NETosis has been further demonstrated to contribute to cancer progression and metastasis (Ronchetti et al., 2021). Interestingly, NETs have been demonstrated to sequester circulating tumor cells to ensure their successful metastatic colonization (Cools-Lartigue et al., 2013). Depletion of neutrophils reduced metastatic dissemination *via* reliving inhibition of cytotoxic T-cell activity (Coffelt et al., 2015). Targeting NETosis using DNase I to dissolve the extracellular DNA or inhibiting protein arginine deiminase 4 (PAD4), an enzyme required for NET formation, using small molecules inhibited cancer growth, metastasis and recurrence in models of breast cancer (Park et al., 2016; Ruiz-Fernandez de Cordoba et al., 2022). Moreover, inhibition of PAD4 using GSK484 sensitizes TNBC xenografts to radiotherapy (Wei et al., 2021) and synergizes with anti-PD1/anti-CTLA dual therapy (Teijeira et al., 2020). Interestingly, NETs were demonstrated to encapsulate tumors cells, preventing physical contact between T-cells and tumor cells (Teijeira et al., 2020; Shinde-Jadhav et al., 2021) and thus may promote T-cell exclusion from the tumor (Figure 4B).

Accumulating evidence suggests that targeting pathway associated with inflammation or cells accumulated at the primary tumor site due to the inflammation can act in synergy with other already approved immunotherapies to lead to collapse of the primary tumor or inhibition of metastasis.

### Development of bispecific antibodies for treatment of breast cancer

Driven by the idea of combinational therapies, bispecific antibodies (bsAbs) that simultaneously bind multiple tumor specific antigens and/or immune cells leading to an anti-tumor immune response have been generated (Dees et al., 2021). Most bsAbs function as CD3^+^ T-cell engagers, that direct T-cells to the tumor epithelium to elicit their anti-tumor function (Dees et al., 2021). Therefore, bsAbs that target T-cells and commonly expressed breast cancer cell surface antigens such as HER2 (Sen et al., 2001), EGFR (Stamm et al., 2019), epithelial cell adhesion molecule (EpCAM) (Kubo et al., 2018), trophoblast cell-surface antigen 2 (Trop2) and carcinoembryonic antigen-related cell adhesion molecule 5 (CEACAM5) (Chang et al., 2017) have been developed to specifically target tumors expressing these markers. Prominently, CD3/HER2 targeting bsAb (Runimotamab) is under clinical investigation for HER2+ breast cancer (NCT03448042). In addition to T-cell engagers, novel natural killer (NK) cell redirectors have been generated through the use of CD16 (Fc receptor expressed on natural killers) to target the breast cancer antigen, mesothelin known as MesobsFab (Del Bano et al., 2019). MesobsFab promoted recruitment and penetration of NK cells into spheroids as well as demonstrating anti-tumor effects in humanized mice (Del Bano et al., 2019).

It is important to note that the success of these antibodies is highly dependent on T-cells being capable of fully infiltrating the tumor. A cold TIME or restriction of T-cells to the margin or stroma may dampen the effect of these antibodies and render the tumors resistant. Efforts aimed at improving the immunogenicity of tumors and immune cell trafficking into the tumor (discussed in “Approaches to Treat Immune Cold Tumors”) may enhance the effects of these therapies. Additionally, bsAbs have demonstrated to work additively with anti-PD1 treatment (Chang et al., 2017) especially because some bsAbs have been demonstrated to induce expression of IFNγ (Sen et al., 2001) which may increase expression of PDL1, suggesting that bsAbs monotherapy may not be suffice due to T-cell exhaustion.

## Targeting the immune compartment at the metastatic site

Nearly all breast cancer deaths are a result of metastasis to essential organs such as brain, lung and liver (Harbeck et al., 2019). Therefore, efforts at both preventing and more importantly treating metastasis at these sites is crucial for improving patient care. Studies using PyMT mice demonstrated that neutrophils accumulate in the lung even during the pre-metastatic stage and are essential for metastatic colonization from the primary tumor site (Wculek and Malanchi, 2015). Additionally, it was demonstrated that tumor derived exosomes carry RNA to the type II alveolar cells of the lung stimulating expression of CXCL1, CXCL2, CXCL5 and CXCL12, through engagement with the toll like three receptor (TLR3), which recruit neutrophils (Liu et al., 2016). TLR3 knockout was sufficient to reduce pulmonary metastasis, suggesting that targeting TLR3 perhaps by a neutralizing antibody (Duffy et al., 2007) may be a viable option at inhibiting metastasis.

Obesity, which is associated with an increase in systemic inflammation is correlated with an increased incidence of metastasis and disease aggressiveness (Carmichael and Bates, 2004). Mouse modelling demonstrated that obesity due to a high fat diet or using the genetic ob/ob model is associated with lung neutrophilia due to upregulation of granulocyte/macrophage (GM)-CSF and IL5 expression (Quail et al., 2017). Profiling of neutrophils demonstrated that obesity was associated with an increase in markers of mobilization and activation and was sufficient to promote lung colonization of PyMT cells from a tail vein assay (Quail et al., 2017) through neutrophil-mediated vasculature permeability (McDowell et al., 2021). Inhibiting NETosis using GSK484 reduced PyMT breast cancer cell extravasation (McDowell et al., 2021) (Figure 4C). Taken together, these studies strongly demonstrate the connection between obesity and lung colonization by cancer cells through obesity mediated priming of the metastatic niche. However, studies demonstrating whether targeting neutrophils (or other immune cells) in an already established metastatic niche are critical for improving patient care for those with metastatic cancer.

Mice harbouring breast-to-liver, but not breast-to-lung metastasis, had a significant increase in low-density neutrophils (LDNs) which are an “immature subset of neutrophils” recruited due to tumor expression of G-CSF (Hsu et al., 2019). Knockdown of G-CSF in liver metastatic cells reduced LDN recruitment and reduced liver metastatic burden (Hsu et al., 2019). This work emphasizes that neutrophils are a heterogenous population of granulocytes and specific subsets may be required for organotropic metastasis.

Accumulating evidence for neutrophils regulating several stages of cancer progression and therapeutic resistance makes them an interesting clinical target. However, to our best knowledge no clinical trial has been established to assess targeting neutrophils or NETosis in breast cancer.

Macrophages represent another interesting target at both the primary and metastatic site. Pulmonary administration of PLX-3397, a CSFR inhibitor reduced metastatic burden in mice harbouring orthotopic 4T1 tumors (Alhudaithi et al., 2020). This was accompanied by a decrease in M2 macrophages and a concomitant increase in M1 macrophages (Alhudaithi et al., 2020). A series of work has identified the immunosuppressive pathway of macrophage stimulating protein (MSP) to be essential for immunosuppression and metastatic outgrowth (Eyob et al., 2013a; Eyob et al., 2013b). MSP is released from the liver as an inactive precursor which becomes activated once cleaved by matriptase (ST14) found on the surface of cancer cells and macrophages (Eyob et al., 2013a). Active MSP binds its receptor, macrophage stimulating one receptor (MST1R/RON), activating a series of downstream signaling in macrophages which are essential for immunosuppression (Eyob et al., 2013a; Eyob et al., 2013b; Lai et al., 2021). MSP overexpression in the PyMT model promoted more aggressive primary tumor behaviour and promoted osteolytic bone metastasis formation (Welm et al., 2007). Targeting RON through genetic knockout or pharmacological inhibition using BMS-777607/ASLAN002 in the PyMT model prevented the formation of overt lung metastasis through upregulation of a series of anti-tumor cytokines; IL12 and IFNγ and downregulation of pro-tumor cytokines; TNFα and IL10 (Eyob et al., 2013a). Inhibition of RON promoted loss of M2 macrophages and induction of cytotoxic and Th1 T-cell activities resulting in reduced growth of pulmonary metastasis (Eyob et al., 2013b; Lai et al., 2021). Moreover, inhibition of RON synergized with anti-CTLA based therapy to inhibit pulmonary metastasis outgrowth (Ekiz et al., 2018). It is important to emphasize that treatment of established micro-metastasis with BMS-777607/ASLAN002, diminished growth of the lesions (Eyob et al., 2013b; Ekiz et al., 2018). This data indicates that inhibition of RON is a viable option for treatment of metastatic disease.

## Conclusions and future directions

It became increasingly clear that novel strategies are required to better classify breast tumors based on a collection of histological, molecular, and immune phenotypes is required to better prognosticate and manage the disease. The breast TIME, at least in the TNBC subtype, is broadly divided into immune hot and immune cold subtypes as well as intermediate subtypes where T-cells are excluded from the tumor nest. While elegant work has been done in understanding the spatial profile of the tumors, functional work identifying pathways to be targeted that lead to abrogation of T-cell exclusion from the tumor nest are still required. Introducing inhibitors that are sufficient to abrogate exclusion of T-cells into a clinical setting may increase the number of responders to T-cell dependent immunotherapy. We are still lacking similar observations to be made in HR+ breast cancer which may allow better stratification of patients and identifying those that respond to immunotherapy versus non-responders. Sophisticated approaches such as single cell sequencing, spatial transcriptomics and multiplex immunohistochemistry and RNA *in situ* hybridization technologies could be leveraged to better understand the complex immunbiology of breast tumors. This may be a particularly interesting avenue especially in the context of ER mutant disease. There is accumulating pre-clinical evidence suggesting that T-cell infiltration and activation through modulation of the STING/adenosine pathways and epigenetic rewiring of the tumor in combination with chemo/radiotherapy may have a clinically positive impact on patient survival and outcome. Oncolytic viruses are an upcoming avenue which may allow for immune mediated elimination of tumors as well as sensitize tumors to immunotherapies. Clinical evaluation of these interventions may lead to introduction of novel clinical interventions. For immune hot tumors, strategies aimed at resolving inflammation due to oncogene activation as well as eliminating immune cells that accumulate due to the chronic inflammation may synergize with ICB as well as inhibit metastasis. Emerging evidence strongly supports that immunotherapy coupled with other precision oncology agents will eventually become part of standard of care for breast cancer management.
